# PGK1 inhibitor CBR-470-1 protects neuronal cells from MPP^+^

**DOI:** 10.18632/aging.103443

**Published:** 2020-07-10

**Authors:** Jinyu Zheng, Jian-liang Zhu, Yufeng Zhang, Hao Zhang, Yu Yang, De-Rong Tang, Jian Sun

**Affiliations:** 1 Department of Neurosurgery, The Affiliated Huai’an Hospital of Xuzhou Medical University, Huai’an, China; 2 Department of Emergency and Intensive Care Unit, The Second Affiliated Hospital of Soochow University, Suzhou, China; 3 Department of Anesthesiology, Huai’an Maternity and Child Clinical College of Xuzhou Medical University, Huai’an, China; 4 Department of Thoracic Surgery, The Affiliated Huaian People’s Hospital of Nanjing Medical University, Huai’an, China

**Keywords:** SH-SY5Y cells, MPP+, CBR-470-1, PGK1, Nrf2

## Abstract

The neurotoxin MPP^+^ (1-methyl-4-phenylpyridinium ion) disrupts mitochondrial function leading to oxidative stress and neuronal death. Here we examine whether activation of the Keap1-Nrf2 cascade can protect SH-SY5Y neuroblastoma cells from MPP^+^-induced cytotoxicity. Treatment of SH-SY5Y cells with CBR-470-1, an inhibitor of the glycolytic enzyme phosphoglycerate kinase 1 (PGK1), leads to methylglyoxal modification of Keap1, Keap1-Nrf2 disassociation, and increased expression of Nrf2 responsive genes. Pretreatment with CBR-470-1 potently attenuated MPP^+^-induced oxidative injury and SH-SY5Y cell apoptosis. CBR-470-1 neuroprotection is dependent upon Nrf2, as Nrf2 shRNA or CRISPR/Cas9-mediated Nrf2 knockout, abolished CBR-470-1-induced SH-SY5Y cytoprotection against MPP^+^. Consistent with these findings, PGK1 depletion or knockout mimicked CBR-470-1-induced actions and rendered SH-SY5Y cells resistant to MPP^+^-induced cytotoxicity. Furthermore, activation of the Nrf2 cascade by CRISPR/Cas9-induced Keap1 knockout protected SH-SY5Y cells from MPP^+^. In Keap1 or PGK1 knockout SH-SY5Y cells,CBR-470-1 failed to offer further cytoprotection against MPP^+^. Collectively PGK1 inhibition by CBR-470-1 protects SH-SY5Y cells from MPP^+^ via activation of the Keap1-Nrf2 cascade.

## INTRODUCTION

In neurons, the disruption of the mitochondrial respiratory chain complex results in reactive oxidative species (ROS) production and oxidative stress. Subsequently, this leads to calcium overload, lipid peroxidation, DNA/protein damage, and eventually neuronal cell death [1]. Inhibitors of the mitochondrial respiratory chain complex, including 1-methyl-4-phenyl-1,2,3,4-tetrahydropyridine (MPTP), 6-hydroxydopamine (6-OHDA), rotenone, are currently being utilized for animal and cellular models of degenerative neuronal disease models [1]. Incubation of dopaminergic (DA) neuronal cells with MPP^+^ (1-methyl-4-phenylpyridinium ion), the MPTP metabolic oxidation byproduct, mimics oxidative injury in Parkinson’s disease [2–4].

The transcription factor Nrf2 exerts a vital defensive activity against oxidative stress. Under resting conditions, Nrf2 targeted for degradation through ubiquitination by binding Keap1, an adaptor protein for the Cul3 ubiquitin ligase complex [5, 6]. Activated Nrf2 protein disassociates from Keap1 and translocates to cell nuclei. After binding to antioxidant response element (ARE) loci, Nrf2 mediates transcription of several key antioxidant enzymes and cytoprotective genes [7–9]. Studies have shown that forced activation of the Nrf2 cascade, through genetic or pharmacological strategies, can protect neuronal cells from oxidative stress [10–12]. Recently, novel Nrf2 activators been identified that can scavenge free radicals and efficiently protect neuronal cells from oxidative injury [13, 14].

Phosphoglycerate kinase 1 (PGK1) is required for ATP synthesis in the process of glycolysis [15, 16]. It catalyzes conversion of 3-phosphoglycerate and ATP from 1,3-diphosphoglycerate and ADP [15, 16]. Recent studies have reported an essential function of PGK1 in inhibiting Keap1-Nrf2 signaling [17]. PGK1 inhibition was found to induce methylglyoxal accumulation that forms a methylimidazole crosslink between proximal cysteine and arginine residues (MICA) in Keap1 [15, 17]. This leads to Keap1 dimerization, Keap1-Nrf2 dissociation and Nrf2 transcriptional activation [15, 17]. Liang et al., demonstrated that PGK1 silencing or knockout (KO) activated Nrf2 signaling to protect human osteoblasts from dexamethasone [18]. Recent studies have characterized a novel PGK1 inhibitor, CBR-470-1 [17]. Here, we demonstrate that PGK1 inhibition by CBR-470-1 protects SH-SY5Y neuronal cells against MPP^+^-induced cytotoxicity through activation of the Keap1-Nrf2 cascade.

## RESULTS

### CBR-470-1 activates Nrf2 signaling cascade in SH-SY5Y cells

**Figure 1:**
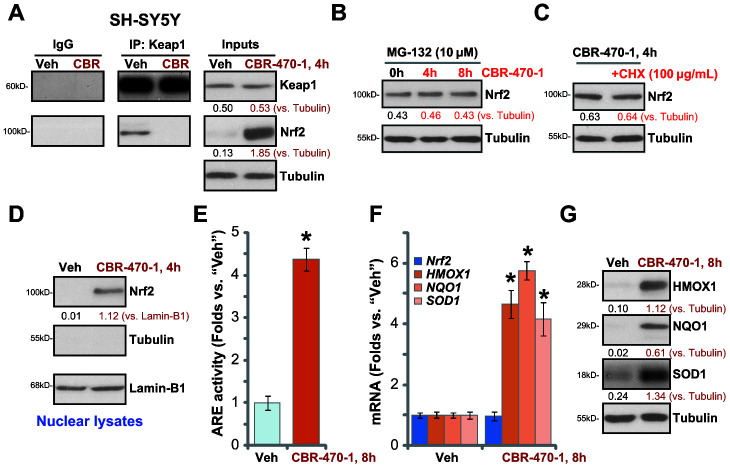
**CBR-470-1 activates Nrf2 signaling cascade in SH-SY5Y cells.** Differentiated SH-SY5Y neuronal cells were treated with CBR-470-1 (10 μM) or the vehicle control (“Veh”), cells were further cultured for the applied time periods, Keap1 immunoprecipitation with Nrf2 was tested by a Co-IP assay (**A**); Nrf2 and Keap1 protein expression in total cell lysates was tested as “Inputs” (**A**). SH-SY5Y neuronal cells were pre-treated with MG-132 (10 μM) for 24h, followed by CBR-470-1 (10 μM) stimulation and cultured for another 4-8h, Nrf2 and Tubulin protein expression in total cell lysates was tested (**B**). SH-SY5Y cells were pretreated with cycloheximide (CHX, 100 μg/mL) for 12h, followed by CBR-470-1 (10 μM) stimulation and cultured for another 4h, Nrf2 and Tubulin protein expression in total cell lysates was shown (**C**). SH-SY5Y neuronal cells were treated with CBR-470-1 (10 μM) or the vehicle control (“Veh”), cells were further cultured for the applied time periods, expression of indicated Keap1-Nrf2 pathway genes was tested by qPCR and Western blotting analyses (**D**, **F**, **G**); Alternatively, cells were harvested and relative ARE luciferase activity was tested, with results normalized to that of vehicle control (**E**). Expression of listed proteins was quantified and normalized to the loading control (**A**–**D**, **G**). Data were expressed as mean ± standard deviation (SD, n=5). * *P*< 0.05 vs. “Veh” cells (**E**, **F**). Experiments were repeated four times with similar results obtained.

CBR-470-1 is a known PGK1 inhibitor. To study whether CBR-470-1 can activate the Nrf2 cascade in DA neuronal cells, we first examined Keap1-Nrf2 association using a co-immunoprecipitation (Co-IP) assay. As demonstrated, Figure 1A, in control vehicle-treated SH-SY5Y cells Keap1 immunoprecipitated with Nrf2, whereasCBR-470-1 treatment (10 μM, 4h) disrupted the interaction. Furthermore, Nrf2 protein levels were significantly increased following Keap1-Nrf2 disassociation in CBR-470-1-treated SH-SY5Y cells, whereas Keap1 expression remained unchanged (Figure 1A).

To examine whether the increased Nrf2 protein levels were the result of decreased proteasomal degradation we treated cells with MG-132, a potent and cell-permeable proteasome inhibitor [19]. CBR-470-1 failed to further increase Nrf2 protein levels after MG-132 pretreatment (Figure 1B), suggesting that CBR-470-1 induced Nrf2 protein elevation by inhibiting its ubiquitin degradation. Furthermore, blocking protein synthesis with cycloheximide (CHX) [20], failed to alter Nrf2 protein levels in CBR-470-1-treated SH-SY5Y cells, suggesting that CBR-470-1 induced Nrf2 protein elevation was not due to protein synthesis. These results support that CBR-470-1 treatment induces Keap1-Nrf2 separation and Nrf2 protein stabilization in SH-SY5Y cells. Testing nuclear fraction proteins, we found that the stabilized Nrf2 protein was enriched in the nuclei of CBR-470-1-treated SH-SY5Y cells (Figure 1D).

We next examined whether CBR-470-1 could induce activation of Nrf2 transcriptional activity.CBR-470-1 robustly increased ARE luciferase activity in SH-SY5Y cells (Figure 1E), promoting expression of ARE-dependent genes, including *HMOX1*, *NQO1* and *SOD1* (Figure 1F). The *Nrf2* mRNA levels were, however, unchanged after CBR-470-1 treatment in SH-SY5Y cells (Figure 1F). Increased protein expression of HMOX1, NQO1 and SOD1 was also detected inCBR-470-1-treated cells (Figure 1G).

### CBR-470-1 inhibits MPP^+^-induced oxidative injury in SH-SY5Y neuronal cells

**Figure 2:**
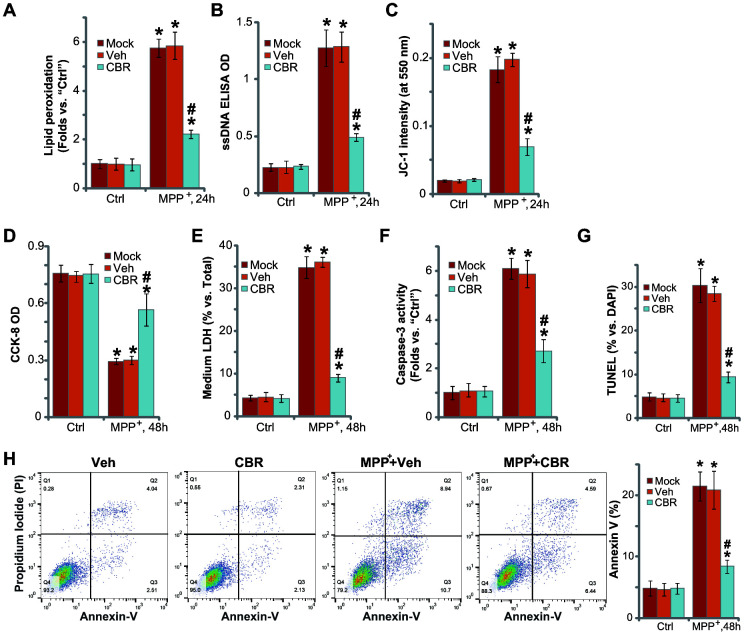
**CBR-470-1 inhibits MPP^+^-induced oxidative injury in SH-SY5Y neuronal cells.** SH-SY5Y neuronal cells were pre-treated for 2h with CBR-470-1 (“CBR”, 10 μM) or the vehicle control (“Veh”), followed by MPP^+^ (3 mM) stimulation, cells were further cultured for applied time periods, relative lipid peroxidation levels (**A**), single strand DNA contents (**B**) and mitochondrial depolarization(JC-1 green fluorescence intensity, (**C**) were tested, and then cell viability and death examined by CCK-8 (**D**) and medium LDH release (**E**) assays, respectively. Cell apoptosis was evaluated by the assays mentioned in the text (**F**–**H**).“Veh” stands for the vehicle control. “Mock” stands for MPP^+^ single treatment (no pretreatment).“Ctrl” stands for no MPP^+^ stimulation. Bars stand for mean ± standard deviation (SD, n=5). * *P*< 0.05 vs. “Ctrl” cells. ^#^*P*< 0.05 vs. “Veh”-pretreated cells. Experiments in this figure were repeated four times, with the similar results obtained.

In line with previous studies [2, 21–23], we found that MPP^+^ induced oxidative injury in SH-SY5Y neuronal cells, causing robust lipid peroxidation (TBAR activity increase, Figure 2A), single strand DNA (ssDNA) accumulation (Figure 2B) and mitochondrial depolarization (JC-1 green fluorescence intensity increase, Figure 2C). Importantly, pretreatment with CBR-470-1(10 μM, 2h) in SH-SY5Y cells attenuated MPP^+^-induced oxidative injury (Figure 2A–2C).

In functional studies, we found that MPP^+^-induced significant viability (CCK-8 OD) reduction (Figure 2D) and cell death (medium LDH release, Figure 2E), which were attenuated with CBR-470-1 pretreatment (Figure 2D, 2E). MPP^+^ induced profound apoptosis, evidenced by increases in caspase-3 activity (Figure 2F), nuclear TUNEL staining (Figure 2G) and Annexin V-positive cell ratio (Figure 2H). Significantly, pretreatment of SH-SY5Y cells with CBR-470-1 ameliorated MPP^+^-induced apoptosis (Figure 2F, 2H). CBR-470-1 treatment alone did not adversely change the function of SH-SY5Y cells (Figure 2A–2H). Collectively, these results show that CBR-470-1 inhibited MPP^+^-induced oxidative injury in SH-SY5Y neuronal cells.

### In SH-SY5Y cells Nrf2 shRNA or KO abolishes CBR-470-1-induced cytoprotection against MPP^+^

**Figure 3:**
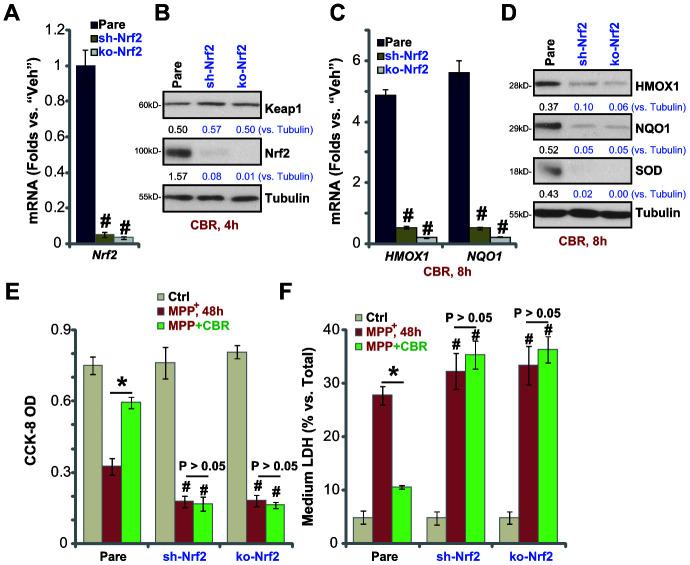
**In SH-SY5Y cells Nrf2 shRNA or KO abolishes CBR-470-1-induced cytoprotection against MPP^+^.** Expression of *Nrf2* mRNA in stable SH-SY5Y neuronal cells with Nrf2 shRNA (“sh-Nrf2”) or a lenti-CRISPR/Cas9-Nrf2 KO construct (“ko-Nrf2”), as well as in the parental control cells (“Pare”), was shown (**A**); Cells were treated with CBR-470-1 (“CBR”, 10 μM) or the vehicle control (“Veh”) for applied time periods, expression of listed mRNAs and proteins was shown (**B**–**D**); Alternatively, cells were pre-treated for 2h with CBR-470-1 (“CBR”, 10 μM) or the vehicle control (“Veh”), followed by MPP^+^ (3 mM) stimulation for 48h, cell viability and death were tested by CCK-8 (**E**) and medium LDH release (**F**) assays, respectively. Expression of listed proteins was quantified and normalized to the loading control (**B**, **D**). Bars stand for mean ± standard deviation (SD, n=5). * *P*< 0.05 (**E**, **F**). ^#^*P*< 0.05 vs. “Pare” cells of same treatment (**A**, **C**, **E**, **F**). Experiments in this figure were repeated four times, with the similar results obtained.

To confirm that the Nrf2 signaling cascade activation is required for CBR-470-1-induced cytoprotection against MPP^+^, a lentiviral Nrf2 shRNA was stably transduced into SH-SY5Y cells, resulting in an over 95% reduction of *Nrf2* mRNA (“sh-Nrf2” cells, Figure 3A). Furthermore, a lenti-CRISPR/Cas9-Nrf2 KO construct was utilized to knockout (KO) Nrf2 in SH-SY5Y cells (“ko-Nrf2” cells, Figure 3A). As shown, CBR-470-1-induced cytosolic accumulation of Nrf2 protein was completely blocked in sh-Nrf2 cells and ko-Nrf2 cells (Figure 3B). Furthermore, CBR-470-1-induced mRNA and protein expression of Nrf2 pathway genes, *HMOX1*, *NQO1* and *SOD*, were completely blocked with Nrf2 silencing or KO (Figure 3C, 3D). Functionally, MPP^+^-induced viability (CCK-8 OD) reduction (Figure 3E) and cell death (testing medium LDH percentage, Figure 3F) were enhanced in sh-Nrf2 cells and ko-Nrf2 cells. Therefore,Nrf2 depletion renders CBR-470-1 ineffective against MPP^+^-induced cytotoxicity in SH-SY5Y cells (Figure 3E, 3F). These results suggest that Nrf2 signaling is required for CBR-470-1-induced cytoprotection in MPP^+^-treated SH-SY5Y cells.

### PGK1 shRNA or KO activates Nrf2 signaling and inhibits MPP^+^-induced cytotoxicity in SH-SY5Y cells

**Figure 4:**
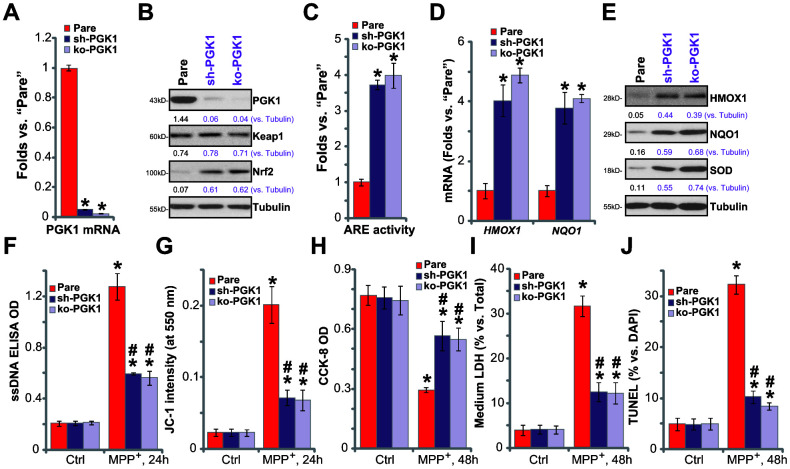
**PGK1 shRNA or KO activates Nrf2 signaling and inhibits MPP^+^-induced cytotoxicity in SH-SY5Y cells.** Expression of listed mRNAs and proteins in the stable SH-SY5Y neuronal cells with PGK1 shRNA (“sh-PGK1”), the lenti-CRISPR/Cas9-PGK1 KO construct (“ko-PGK1”), as well as in the parental control cells (“Pare”), was tested by qPCR (**A**, **D**) and Western blotting (**B**, **E**) analyses, with the relative ARE luciferase activity tested as well (**C**); Alternatively, cells were treated with MPP^+^ (3 mM) for 24-48h, single strand DNA contents (**F**) and mitochondrial depolarization (JC-1 green fluorescence intensity, **G**) were tested; Cell viability, death and apoptosis were tested by CCK-8 (**H**), medium LDH release (**I**) and nuclear TUNEL staining (**J**) assays, respectively. Expression of listed proteins was quantified and normalized to the loading control (**B**, **E**). Bars stand for mean ± standard deviation (SD, n=5). * *P*< 0.05 vs. “Pare” cells (**A**, **C**, **D**). * *P*< 0.05 vs. “Pare” cells with “Ctrl” treatment (**F**–**J**).^#^*P*< 0.05 vs. MPP^+^-treated “Pare” cells (**F**–**J**). Experiments in this figure were repeated four times, with the similar results obtained.

If the neuroprotective effects of CBR-470-1 are elicited through inhibition of PGK1,PGK1 silencing or depletion would be predicted to similarly induce Nrf2 cascade activation in SH-SY5Y neuronal cells. To test this, differentiated SH-SY5Y cells were transduced with the lentiviral PGK1 shRNA (“sh-PGK1”, from Dr. Tan [18]), and cells selected by puromycin to establish stable cells (“sh-PGK1” cells). Additionally, a CRISPR/Cas9 gene-editing strategy, as reported [18], was applied to KO PGK1 in SH-SY5Y cells (“ko-PGK1” cells). As shown, expression of *PGK1* mRNA (Figure 4A) and protein (Figure 4A) decreased by over 95% in both sh-PGK1 cells and ko-PGK1 cells. Nrf2 protein accumulated with PGK1 silencing or KO (Figure 4B), leading to increased ARE luciferase activity (Figure 4C) and expression of Nrf2 pathway genes (*HMOX1*, *NQO1* and *SOD*, Figure 4D, 4E).

PGK1 depletion can activate Nrf2 signaling in SH-SY5Ycells, mimicking CBR-470-1-induced activity. Functional studies demonstrate that in sh-PGK1 cells and ko-PGK1 cells, MPP^+^-induced ssDNA accumulation (Figure 4F) and mitochondrial depolarization (Figure 4G) were largely attenuated (vs. the parental control cells). These results suggest that PGK1 silencing or KO ameliorated MPP^+^-induced oxidative stress in SH-SY5Y cells. Additionally, with PGK1 silencing or KO SH-SY5Y cells were protected from MPP^+^, presenting with significantly inhibited viability reduction (Figure 4H), cell death (Figure 4I) and apoptosis (Figure 4J), when compared to parental control cells. Therefore, PGK1 silencing or KO inhibited MPP^+^-induced oxidative stress and cytotoxicity in SH-SY5Y cells.

### In Keap1-KO or PGK1-KO SH-SY5Y cells CBR-470-1 fails to offer further cytoprotection against MPP^+^

**Figure 5:**
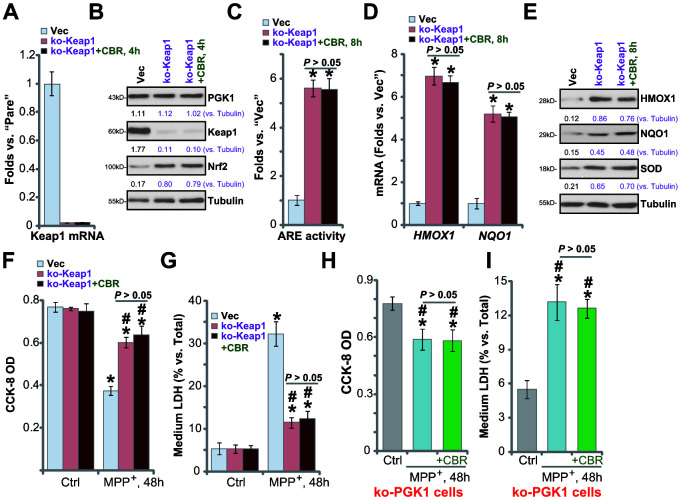
**In Keap1-KO or PGK1-KO SH-SY5Y cells CBR-470-1 fails to offer further cytoprotection against MPP^+^.** Expression of listed mRNAs and proteins in empty vector (“Vec”)-expressing SH-SY5Y cells or stable SH-SY5Y cells with a lenti-CRISPR/Cas9-Keap1 KO construct (“ko-Peak1”), treated with or without CBR-470-1 (“CBR”, 10 μM, for 4-8h), was tested by qPCR (**A**, **D**) and Western blotting (**B**, **E**) assays, with the relative ARE luciferase activity tested as well (**C**); Alternatively, cells were treated with MPP^+^ (3 mM) for 48h, cell viability and death were tested by CCK-8 (**F**) and medium LDH release (**G**) assays, respectively. The ko-PGK1 cells were pre-treated for 2h with CBR-470-1 (“+CBR”, 10 μM), followed by MPP^+^ (3 mM) stimulation for 48h, with cell viability (**H**) and death (**I**) tested similarly. Expression of listed proteins was quantified and normalized to the loading control (**B**, **E**). Bars stand for mean ± standard deviation (SD, n=5). * *P*< 0.05 vs. “Vec” cells (**A**, **C**, **D**). * *P*< 0.05 vs. “Vec” cells with Ctrl treatment (**F**, **G**). ^#^*P*< 0.05 vs. MPP^+^-treated “Vec” cells (**F**, **G**). * *P*< 0.05 vs. “Ctrl” treatment (**H**, **I**). Experiments in this figure were repeated four times, with the similar results obtained.

It is predicted that Keap1 depletion should also activate the Nrf2 cascade, protecting SH-SY5Y cells from MPP^+^-induced cytotoxicity. A CRISPR/Cas9 Keap1-KO construct (from Dr. Zhen at Soochow University [24]) was transduced into SH-SY5Y cells. Following FACS-mediated GFP sorting and Keap1 KO screen, the stable and monoclonal Keap1 KO SH-SY5Y cells (“ko-Keap1”) were established, showing completely depleted *Keap1* mRNA (Figure 5A) and protein (Figure 5B). Keap1 KO resulted in Nrf2 protein stabilization and accumulation (Figure 5B), increased ARE activity (Figure 5C), and expression of Nrf2 pathway genes (*HMOX1*, *NQO1* and *SOD*) (Figure 5D, 5E), without altering PGK1 expression (Figure 5B). Therefore, Keap1 KO induced robust Nrf2 cascade activation in SH-SY5Y cells.

Significantly, in ko-Keap1 cells, CBR-470-1 did not further increase activation of Nrf2 cascade (Figure 5B–5E). The ko-Keap1 SH-SY5Y cells were resistant to MPP^+^ stimulation, with little viability reduction (Figure 5F, *P*< 0.05 vs. vector control cells) and cell death (Figure 5G, *P*< 0.05 vs. vector control cells) after MPP^+^ treatment. In ko-Keap1 SH-SY5Y cells, CBR-470-1 failed to offer additive cytoprotection against MPP^+^ (Figure 5F, 5G). These results show that Keap1 KO can mimic CBR-470-1-induced cytoprotection against MPP^+^ in SH-SY5Y cells, further supporting that the Keap1-Nrf2 cascade is required for CBR-470-1-induced actions. Further supporting this conclusion, we found that in the ko-PGK1 SH-SY5Y cells (see Figure 4) addition of CBR-470-1 failed to offer further cytoprotection against MPP^+^ (Figure 5H, 5I).

## DISCUSSION

Recent studies have reported an important link between glycolysis and Nrf2 cascade signaling through reactive metabolite-induced posttranslational modification of Keap1 [17, 18]. Following glycolysis, the accumulated reactive dicarbonyl metabolite methylglyoxal induces Nrf2 signaling activation [17, 18]. Methylglyoxal accumulation leads to the formation of a methylimidazole crosslink between cysteine residues and proximal arginine residues between two monomeric Keap1 [17, 18]. This results in Keap1 dimerization, Keap1-Nrf2 disassociation, and Nrf2 cascade activation [17, 18].

Studies have shown that PGK1 inhibition or silencing leads to the accumulation of methylglyoxal and subsequent activation of the Keap1-Nrf2 cascade [17, 18]. In the present study we show that CBR-470-1 activates the Nrf2 cascade in SH-SY5Y neuroblastoma cells, causing disassociation of the Keap1-Nrf2 complex, cytosol Nrf2 protein stabilization and nuclear translocation, followed by increased expression of Nrf2 pathway genes (*HMOX1*, *NQO1* and *SOD1*).

MPP^+^ disrupts the mitochondrial respiratory chain, causing ROS production and oxidative injury, eventually leading to neuronal cell death [22, 25, 26]. Forced activation of the Nrf2 cascade, using genetic or pharmacological strategies, protects DA neuronal cells from MPP^+^. Kabaria et al., found that microRNA-7 silenced Keap1 to activate Nrf2 cascade and to protect SH-SY5Y cells from MPP^+^ [22]. Zhu et al., discovered that SC79, a novel Akt activator [27–29], protected SH-SY5Y cells from MPP^+^ by activating the Akt-Nrf2 cascade [2]. Funakohi-Tago et al., discovered that activation of the Nrf2 cascade by hydroxytyrosol butyrate inhibited 6-OHDA-induced apoptosis in SH-SY5Y cells [10]. In the present study we demonstrate that CBR-470-1 potently ameliorated MPP^+^-induced oxidative injury and cell apoptosis in SH-SY5Y cells. Nrf2 depletion reversed CBR-470-1-induced SH-SY5Y cytoprotection against MPP^+^. Thus, activation of Nrf2 by CBR-470-1 alleviated MPP^+^-induced oxidative injury in SH-SY5Y neuronal cells.

Our results further support that PGK1 inhibition is the main mechanism of CBR-470-1-induced Nrf2 cascade activation and SH-SY5Y cell cytoprotection against MPP^+^. Mimicking CBR-470-1’s activity, PGK1 depletion induced significant Nrf2 cascade activation. Functional studies demonstrated that SH-SY5Y cells with PGK1 silencing or KO were protected from MPP^+^-induced cytotoxicity and apoptosis. In PGK1-depleted SH-SY5Y cells, CBR-470-1 failed to induce further Nrf2 cascade activation or offer additional cytoprotection against MPP^+^. These results suggest that PGK1 inhibition by CBR-470-1 activated Nrf2 signaling cascade, thereby protectingSH-SY5Y neuronal cells from MPP^+^. To further support our hypothesis, we show that forced activation of the Nrf2 cascade by Keap1 KO similarly inhibited MPP^+^-induced-cytotoxicity in SH-SY5Y cells. Furthermore, CBR-470-1 was once again completely ineffective on MPP^+^-induced actions in Keap1 KO cells with sustained Nrf2 activation.

## CONCLUSIONS

We discovered that CBR-470-1 activated the Nrf2 cascade to protect SH-SY5Y cells from MPP^+^-induced oxidative injury, suggesting that CBR-470-1 could be promising drug for neuron protection.

## MATERIALS AND METHODS

### Chemicals, reagents and antibodies

CBR-470-1 was synthesized by Shanghai Rui-lu Biotech (Shanghai, China) based on a previously-described protocol [17]. MPP^+^ was purchased from Sigma-Aldrich (St. Louis, Mo). All the antibodies utilized in the present study were purchased from Cell Signaling Tech (Danvers, MA). Fetal bovine serum (FBS), antibiotics and other cell culture reagents were from Gibco Co. (Suzhou, China). Primers and reagents for RNA studies were provided by Shanghai Genechem Co. (Shanghai, China).

### SH-SY5Y cells

SH-SY5Y DA neuronal cells were provided by Dr. Zhang at Soochow University [2], cultured using a previously-described protocol [26]. For cell differentiation, SH-SY5Y cells were first cultured in complete DMEM (5% FBS) plus 10 μM all-trans retinoic acid (RA) for three days. Cells were then cultured in Neurobasal-A medium (NB) (minus phenol red, Invitrogen), supplemented with 1% L-glutamine (200 mM), 1% N-2 supplement (Invitrogen), and 1% P/S. Human BDNF (at 50 ng/mL) was added shortly. After another three days, SH-SY5Y cells were differentiated. The number of neurites in each differentiated SH-SY5Y cells is 4-5, and the average length of the neurites is close to 15 μm.

### Lactate dehydrogenase (LDH) studies

The differentiated SH-SY5Y cells were cultured onto six well-tissue plates (at 1×10^5^ cells per well). After the indicated MPP^+^ treatment, medium LDH contents and total LDH contents were examined by a two-step LDH detection kit (Promega, Shanghai, China). The medium LDH was normalized (% *vs.* total LDH).

### Cell viability

The differentiated SH-SY5Y cells were cultured onto six well-tissue plates (at 1×10^5^ cells per well). Following the applied MPP^+^ treatment, cell viability was quantified via a cell counting kit-8 (CCK-8) assay (Dojindo Molecular Technologies, Kumamoto, Japan), and its optical density (OD) values tested at 550 nm.

### Western blotting and co-immunoprecipitation (co-IP)

The detailed protocols of Western blotting were previously reported [30, 31]. In brief, lysate proteins were separated by SDS-PAGE gels [32], transferred to PVDF blots(Millipore, Shanghai, China). The blots were blocked and incubated with the designated primary and secondary antibodies. An ECL reagent kit (Pierce, Shanghai, China) was applied to detect the protein band under X-ray films. Data quantification was carried out by an ImageJ software (NIH). For the co-IP studies, the quantified protein lysates (1, 000 μg for each treatment) were pre-cleared and incubated with anti-Keap1 antibody [33]. Keap1-Nrf2 complex was captured by the G-Sepharose (“Beads”, Sigma), tested by Western blotting. Testing nuclear fraction lysates was described in our previous studies [30, 31]

### Caspase-3 activity

SH-SY5Y cells were cultured onto six well-tissue plates (at 1×10^5^ cells per well). Following the applied MPP^+^ treatment, the caspase-3 activity was tested by a commercial fluorometric caspase-3 assay kit (Beyotime Biotechnology, Wuxi, China) [34], using the previously-described protocol [31].

### Cell apoptosis analyses

The detailed protocols for TUNEL [terminal deoxynucleotidyl transferase (TdT)-mediated dUTP nick end labeling] nuclear staining assay and Annexin V fluorescence-activated cell sorting (FACS) were described in detail previously [35, 36]. For TUNEL assays, TUNEL percentage (% *vs.* DAPI staining) of 500 cells per treatment in five random views (1: 200 magnification) was calculated.

### Lipid peroxidation quantification

As described elsewhere [37], the cellular lipid peroxidation levels were examined by using a thiobarbituric acid reactive substances (TBAR) activity assay kit [38].

### Single strand DNA (ssDNA) ELISA

The differentiated SH-SY5Y cells were cultured onto six well-tissue plates (at 1×10^5^ cells per well). After the indicated MPP^+^ treatment, forty μg (40 μg) total cell lysates of each treatment were analyzed by a ssDNA ELISA kit (Roche, Basel, Switzerland) to quantify DNA fragmentations. The ssDNA ELISA absorbance was tested at 405 nm.

### ARE reporter assay

The differentiated SH-SY5Y cells were cultured onto six well-tissue plates (at 1×10^5^ cells per well), and transfected with an ARE-inducible firefly luciferase vector (from Dr. Jiang at Nanjing Medical University [39]). The transfected cells were subjected to applied treatment, and the luciferase activity tested by a luminescence instrument.

### Mitochondrial depolarization

The differentiated SH-SY5Y cells were cultured onto six well-tissue plates (at 0.8×10^5^ cells per well). Following the applied MPP^+^ treatment for 24h, the fluorescence dye JC-1 (Thermo-Fisher Invitrogen, Shanghai, China) was added to detect mitochondrial depolarization (“ΔΨ”). JC-1 will aggregate in the mitochondria, forming green monomers with mitochondrial depolarization in cells with oxidative injury. JC-1 fluorescence absorbance was recorded at 550 nm.

### The quantitative real-time reverse transcriptase polymerase chain reaction (qPCR)

The detailed protocols of qPCR were described previously [30, 31]. Total cellular RNA was extracted by TRIzol reagents (Sigma), with reverse transcription and qPCR performed using a TOYOBO ReverTra Ace qPCR kit (Tokyo, Japan) and under the ABI Prism 7500H qPCR Instrument (Applied Biosystems, Foster City, CA). The melt curve analysis was performed, and a 2^−ΔΔ*C*t^ method applied for quantification of targeted mRNAs using *GAPDH* as the reference gene. The primers for Nrf2 pathway gens, including *heme oxygenase-1* (*HMOX1*),*NADPH: quinone acceptor oxidoreductase 1* (*NQO1*), and *superoxide dismutase* (*SOD*), as well as *Nrf2*, *Keap1* and *PGK1*, were provided by Dr. Tan at Sino-Japanese Friendship Hospital [18].

### shRNA

PGK1 shRNA-expressing lentivirus and Nrf2 shRNA-expressing lentivirus were provided by Dr. Tan at Sino-Japanese Friendship Hospital [18], that was individually transduced to SH-SY5Y cells (cultured in polybrene-containing complete medium). Following selection by puromycin the stable cells were established, with PGK1 or Nrf2 silencing (over 95% knockdown efficiency) confirmed by qPCR and Western blotting analyses. Control SH-SY5Y cells were transduced with lentiviral scramble control shRNA (“sh-C”).

### PGK1 knockout (KO)

A CRISPR/Cas9 PX458-PGK1-KO-GFP construct (from Dr. Tan at Sino-Japanese Friendship Hospital [18]) was transduced to SH-SY5Y cells by Lipofectamine 2000 (Thermo-Fisher, Invitrogen, Shanghai, China). FACS was then applied to sort GFP-positive cells, with single PGK1 KO cells distributed and verified. Control cells were transduced with control construct (from Dr. Tan at Sino-Japanese Friendship Hospital [18]).

### CRISPR/Cas9-induced KO of Nrf2 or Keap1

The monoclonal SH-SY5Y cells with a lentiCRISPR-GFP-Nrf2-puro KO construct (Nrf2-KO SH-SY5Y cells), as well as the control cells with empty vector (“Vec”), were provided by Dr. Di at Wannan Medical College [40]. A lentiCRISPR-Keap1-KO-puro-GFP construct, provided by Dr. Zhen at Soochow University [24], was transduced to SH-SY5Y cells. Using FACS, GFP-positive cells were sorted and distributed to 24-well plates. Keap1-KO was screened and stable monoclonal cells established, with Keap1 KO verified by qPCR and Western blotting analyses.

### Statistics

Data were presented as mean ±standard deviation (SD). Statistical differences were analyzed by one-way analysis of variance (ANOVA) followed by multiple comparisons performed with post hoc Bonferroni test (SPSS). A two-tailed unpaired T test (Excel 2007) was utilized to examine significance between two treatment groups. Values of ***P***< 0.05 were considered statistically significant. Values of ***P***> 0.05 stand for no significant difference.
